# Point-of-care motion capture and biomechanical assessment improve clinical utility of dynamic balance testing for lower extremity osteoarthritis

**DOI:** 10.1371/journal.pdig.0000068

**Published:** 2022-07-07

**Authors:** Ryan T. Halvorson, Francine T. Castillo, Fayyaz Ahamed, Karim Khattab, Aaron Scheffler, Robert P. Matthew, Jeffrey Lotz, Thomas P. Vail, Brian T. Feeley, Jeannie F. Bailey

**Affiliations:** 1 Department of Orthopaedic Surgery, University of California San Francisco, United States of America; 2 School of Medicine, University of California San Francisco, United States of America; 3 Department of Epidemiology and Biostatistics, University of California San Francisco, United States of America; 4 Department of Physical Therapy and Rehabilitation, University of California San Francisco, United States of America; cereneo Foundation, SWITZERLAND

## Abstract

Musculoskeletal conditions impede patient biomechanical function. However, clinicians rely on subjective functional assessments with poor test characteristics for biomechanical outcomes because more advanced assessments are impractical in the ambulatory care setting. Using markerless motion capture (MMC) in clinic to record time-series joint position data, we implemented a spatiotemporal assessment of patient kinematics during lower extremity functional testing to evaluate whether kinematic models could identify disease states beyond conventional clinical scoring. 213 trials of the star excursion balance test (SEBT) were recorded by 36 subjects during routine ambulatory clinic visits using both MMC technology and conventional clinician scoring. Conventional clinical scoring failed to distinguish patients with symptomatic lower extremity osteoarthritis (OA) from healthy controls in each component of the assessment. However, principal component analysis of shape models generated from MMC recordings revealed significant differences in subject posture between the OA and control cohorts for six of the eight components. Additionally, time-series models of subject posture change over time revealed distinct movement patterns and reduced overall postural change in the OA cohort compared to the controls. Finally, a novel metric quantifying postural control was derived from subject specific kinematic models and was shown to distinguish OA (1.69), asymptomatic postoperative (1.27), and control (1.23) cohorts (p = 0.0025) and to correlate with patient-reported OA symptom severity (R = -0.72, p = 0.018). Time series motion data have superior discriminative validity and clinical utility than conventional functional assessments in the case of the SEBT. Novel spatiotemporal assessment approaches can enable routine in-clinic collection of objective patient-specific biomechanical data for clinical decision-making and monitoring recovery.

## Author summary

Osteoarthritis (OA) is a leading cause of disability in the United States. Despite the relevance of biomechanical function as a marker of disease severity and as a target for therapeutic interventions, clinical assessments of biomechanical function are significantly limited by clinician subjectivity and poor test characteristics while more advanced methods are not feasible due to the need for specialized equipment and trained personnel. Coupling a single markerless motion capture camera with statistical modeling of posture change, we developed a practical system to perform advanced biomechanical assessments of lower extremity function during routine clinic visits. To validate our system, OA patients and healthy controls were assessed performing a functional balance task by clinicians according to conventional scoring and separately by our motion capture system using kinematic posture modeling. Although clinical scoring failed to distinguish OA patients and healthy controls, our kinematic modeling and dimensionality reduction techniques identified significant differences in both subject posture and motion trajectories throughout the assessment. Furthermore, OA patients reporting more severe symptoms exhibited worse postural control. Our results imply that novel motion capture approaches can enable routine in-clinic collection of objective patient-specific biomechanical data for clinical decision-making and monitoring recovery.

## Introduction

Musculoskeletal conditions impede patient biomechanical function. However, we continue to rely on a largely subjective musculoskeletal physical examination for biomechanical outcomes that is limited by poor accuracy, reliability, and repeatability [1–3]. The rapid development of motion capture technologies has enabled significant advancements in the objectivity and accuracy of the assessment of musculoskeletal health. However, these technologies often require high-cost motion capture systems and trained personnel operating in a specialized, pre-calibrated testing environment, with subjects having to wear multiple markers to aid computer vision. These factors have limited the widespread adoption of these technologies in clinical settings and complicated the development of large clinical datasets that will be necessary to estimate population and disease specific distributions. Recent commercially available markerless motion capture (MMC) cameras have been developed that do not require specialized workspaces and equipment, generating a more pragmatic solution for assessment in clinics and other settings [4–6].

One example of a functional test for the lower extremity is the star excursion balance test (SEBT). The SEBT is an assessment of dynamic postural control during which a subject balances on one leg and maximally reaches in each of eight directions with the contralateral leg without falling or shifting weight to the reaching leg. The conventional SEBT output score is the distance reached in each direction. The SEBT has been validated and utilized in various patient populations to study conditions such as osteoarthritis (OA), patellofemoral pain, ankle instability, ligament reconstructions, lower back pain, and athletic injuries [7–11]. SEBT scores have been shown to have discriminative validity between disease states and to have predictive validity for athletic injuries [12–15].

However, administration of the SEBT is prone to error as all eight scores must be recorded manually, with reported intra-rater reliability ranging from 0.67–0.97 and inter-rater reliability ranging from 0.32–0.96 [16–19]. To address these limitations, others have attempted to validate administration using motion capture technology. Kanko et al used traditional motion capture in a specialized setting to administer SEBT to 37 knee OA patients and observed high correlations with manual measurements [8]. Eltouhky compared traditional motion capture to a MMC system for ten patients during a simplified version of the SEBT and observed excellent agreement and consistency in lower extremity joint angles and reach distances [20]. These approaches have primarily confirmed the accuracy and reproducibility of motion capture in administration of the SEBT, but report only conventional kinematic outcomes (e.g. peak joint angles) and do not demonstrate the clinical utility of more advanced statistical methods (e.g. dimensionality reduction).

In contrast to static postural control which refers to the ability to maintain balance in a specific posture, dynamic postural control reflects the ability to balance over the course of completing a task. The conventional SEBT output metric, reach distances, serve as a proxy for dynamic stance leg stability under the assumption that greater postural control allows for greater reach distances. However, no direct assessment of the trunk or stance leg is recorded in the conventional SEBT and there is no temporal component since the maximal reach is measured at only one time point during the assessment. Because most activities of daily living (e.g. gait, standing from a chair, transitioning between postures) are inherently dynamic, an assessment based on peak values alone without time series information risks ignoring relevant clinical biomechanical information [21].

**Fig 1:**
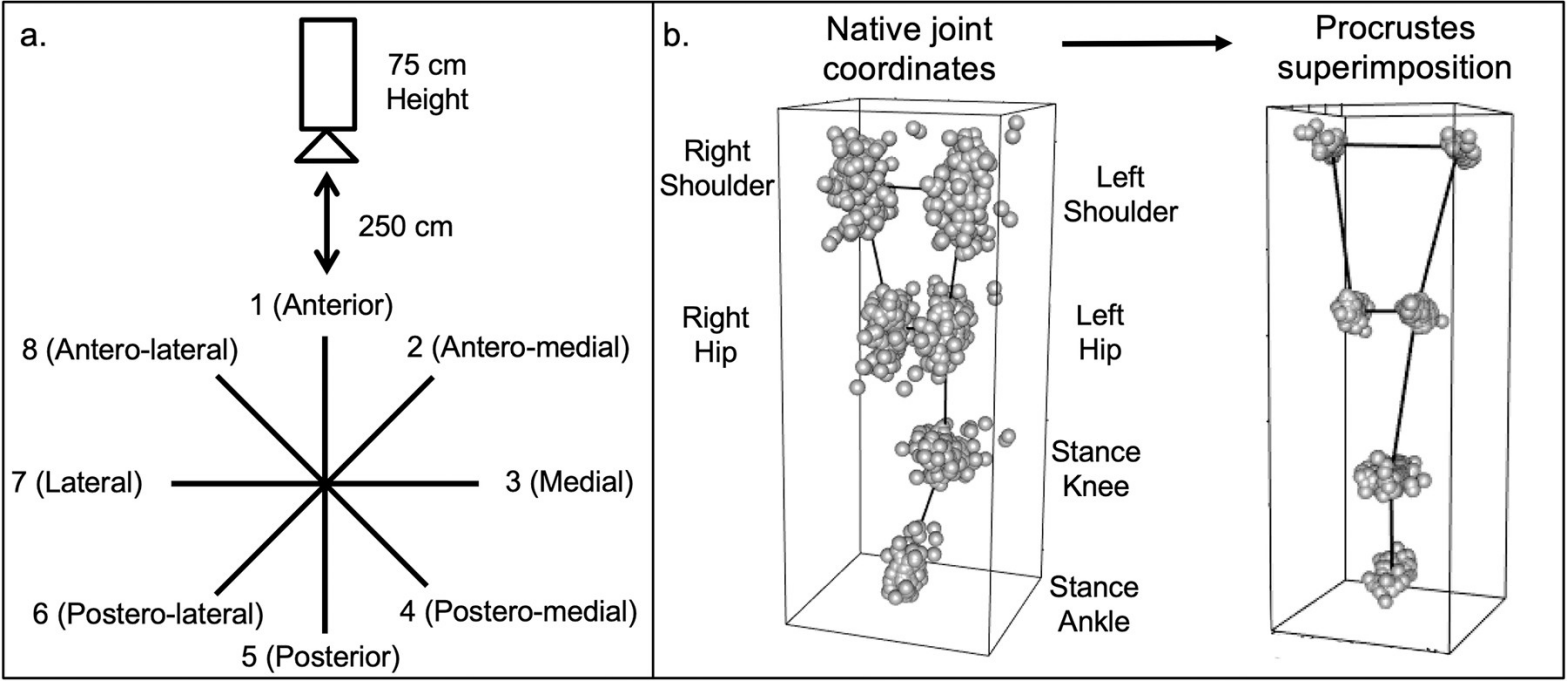
Star Excursion Balance Test Reach Directions and Visual Overview of Generalized Procrustes Analysis. a. Illustration of SEBT configuration, floor grid, and camera orientation. During the assessment, subjects balance on the center of the grid and attempt to reach as far as possible with the other toe in each of the eight noted directions. B. Visual overview of Generalized Procrustes Analysis with Procrustes superimposition. The raw joint coordinates (right) are transformed with standardization for scaling, rotation, and translation. The resulting superimposed coordinates are on the left.

In this study, we assessed the accuracy of MMC for SEBT in the clinical setting. We performed conventional SEBT assessments of three groups of subjects–healthy controls, patients with lower extremity OA, and asymptomatic patients who previously underwent orthopaedic surgical procedures of the lower extremity. Based on the limitations of the conventional SEBT output, we used MMC to quantify and compare movement patterns of the stance leg and trunk using statistical shape modeling (Fig 1). Finally, we propose a novel kinematic deviation index (KDI) to approximate overall postural control during the assessment and demonstrate both its discriminative ability and its relationship with patient reported health measures in a cohort of OA patients. We hypothesize that spatiotemporal assessment of the stance leg and trunk during SEBT using MMC in a clinical setting could detect underlying differences in posture kinematics between different disease states beyond conventional SEBT reach distances.

## Results

### Study population

A total of 213 SEBT trials were performed on 71 legs by 36 subjects during routine ambulatory clinic visits (Table 1). The average subject was 45.7 years old (SD 17.9), with a height of 173.5 cm (SD 10.03) and BMI 27.5 kg/m^2^ (SD 4.25). Of the 36 subjects, 19 were healthy controls, eight had lower extremity OA (three with knee predominant symptoms, and five with hip predominant symptoms), and 9 were asymptomatic postoperative patients undergoing routine follow up. Among the patients in the OA group, the average hip disability and osteoarthritis outcome score (HOOS) and knee injury and osteoarthritis outcome score (KOOS) scores were 37.50 (SD 18.97). There was a significant difference in age between groups (p < 0.05), but there was no relationship between groups and sex, height, weight, or BMI.

**Table 1:** Summary of study population.

Group	N	Age (SD)	Male (%)	Height (SD)	Weight (SD)	BMI (SD)	HOOS / KOOS (SD)
Controls	19	37.11 (11.82)	13 (68.4)	174.02 (9.42)	82.74 (14.96)	27.23 (3.99)	
OA	8	70.38 (9.26)	5 (62.5)	177.96 (10.05)	86.50 (18.26)	26.93 (3.06)	37.50 (18.97)
Post-operative	9	42.00 (14.2)	5 (55.5)	168.43 (9.02)	80.69 (13.28)	28.61 (5.34)	
Totals	36	45.72 (17.92)	23	173.50 (10.03)	83.06 (15.5)	27.51 (4.25)	

N, number of participants. SD, standard deviation. OA, lower extremity osteoarthritis. Post-op, asymptomatic post-operative patients. BMI, body mass index. HOOS, Hip disability and osteoarthritis outcome score. KOOS, Knee injury and OA outcome score.

### Accuracy of conventional SEBT with MMC

Repeated measures correlation was employed to compare the relationship between manually measured reach distances and those obtained through MMC. The following are the correlation coefficients for each reach direction: reach one (0.72, 95% CI 0.63–0.79), reach two (0.78, 95% CI 0.7–0.83), reach three (0.64, 95% CI 0.53–0.73), reach four (0.69, 95% CI 0.59–0.77), reach five (0.37, 95% CI 0.22–0.5), reach six (0.34, 95% CI 0.18–0.48), reach seven (0.17, 95% CI -0.01–0.33) and reach eight (0.5, 95% CI 0.36–0.61).

### Distinguishing subject groups using conventional SEBT

In repeated-measures mixed methods linear regression models, leg length-normalized reach distances failed to distinguish OA patients and post-operative patients from controls in any of the eight reach directions (Table 2). Age was modeled as a fixed effect and had a small but statistically significant association with reach distance in direction two (95% CI -0.04–0.00), three (95% CI -0.05–0.01), four (95% CI -0.05–0.00), five (95% CI -0.05–0.00), six (95% CI -0.05–0.02), seven (95% CI -0.02–0.00, and eight (95% CI -0.04–0.00). Sex and affected limb status were also modeled as fixed effects but were not associated with reach distances in any direction.

**Table 2:** Repeated-measures mixed methods linear regression models to predict reach distances.

		Fixed Effects					
		Group					
Direction		Control	OA	Post-operative	Age	Sex (Male)	Affected Limb
1	Estimate	Ref	0.99	3.84	-0.13	0.61	-1.63
	95% CI		-0.70–0.90	-0.31–1.08	-0.03–0.00	-0.42–0.54	-0.82–0.5
	P value		0.81	0.3	0.17	0.81	0.64
2	Estimate	Ref	-0.28	-1.56	-0.21	2.26	2.41
	95% CI		-0.86–0.80	-0.88–0.57	-0.04–0.00	-0.27–0.72	-0.45–0.93
	P value		0.95	0.68	**0.03**	0.39	0.51
3	Estimate	Ref	1.05	-0.7	-0.31	6.72	1.97
	95% CI		-0.82–1.02	-0.87–0.73	-0.05–0.01	0.12–1.22	-0.56–0.96
	P value		0.83	0.87	0.003*	0.02	0.62
4	Estimate	Ref	-8.53	-1.74	-0.25	5.96	2.6
	95% CI		-1.81–0.10	-1.01–0.66	-0.05–0.00	0.03–1.17	-0.53–1.05
	P value		0.1	0.07	**0.02**	0.05	0.53
5	Estimate	Ref	-4.88	1.01	-0.27	1.71	1.12
	95% CI		-1.52–0.55	-0.8–1	-0.05–0.00	-0.45–0.79	-0.75–0.97
	P value		0.38	0.83	**0.03**	0.6	0.81
6	Estimate	Ref	-4.39	-3.36	-0.37	0.09	-0.84
	95% CI		-1.22–0.34	-1.01–0.34	-0.05–0.02	-0.45–0.47	-0.74–0.57
	P value		0.29	0.35	< 0.001*	0.97	0.81
7	Estimate	Ref	-2.4	-1.74	-0.14	0.02	-0.13
	95% CI		-0.76–0.27	-0.6–0.25	-0.02–0.00	-0.3–0.3	-0.46–0.44
	P value		0.38	0.45	**0.02**	0.99	0.96
8	Estimate	Ref	-3.14	0.48	-0.21	-3.94	-0.84
	95% CI		-1.08–0.46	-0.62–0.72	-0.04–0.00	-0.85–0.07	-0.74–0.57
	P value		0.44	0.89	**0.02**	0.11	0.81

OA, osteoarthritis group. P values < 0.05 are bolded. P values below the Bonferroni corrected threshold of 0.00625 are indicated with an asterisk.

### Distinguishing subject groups using three-dimensional posture at maximal reach

Three-dimensional coordinates for the stance ankle, stance knee, bilateral hips, and bilateral shoulders were filtered and transformed in a generalized Procrustes analysis (GPA) to normalize body size, translation, and rotation. There was a significant relationship between posture at the time of maximum reach and disease state in six of the eight reach directions after controlling for the effects of age, sex, and affected leg: reach one (p = 0.003, F = 3.25), reach two (p = 0.001, F = 6.89), reach three (p = 0.001, F = 6.4), reach four (p = 0.003, F = 6.41), reach five (p = 0.002, F = 5.39), and reach six (p = 0.002, F = 5.2) (Table 3). No association was observed for directions seven (p = 0.35, F = 1.09) or eight (p = 0.15, F = 1.57).

**Table 3:** Relationship between posture at maximal reach and disease state, controlling for age, sex, and affected leg after Procrustes ANOVA.

	Group				Age				Sex				Affected Leg			
D	SS	R^2^	F	p	SS	R^2^	F	p	SS	R^2^	F	p	SS	R^2^	F	p
1	0.05	0.09	3.25	0.003*	0.004	0.01	0.46	0.752	0.01	0.02	1.27	0.278	0.002	0.003	0.23	0.913
2	0.14	0.16	6.89	0.001*	0.02	0.03	2.32	0.069	0.04	0.05	3.91	**0.023**	0.001	0.001	0.09	0.991
3	0.17	0.14	6.4	0.001*	0.05	0.04	3.81	**0.018**	0.1	0.08	7.06	0.007*	0.001	0.001	0.07	0.993
4	0.22	0.14	6.41	0.003*	0.11	0.07	6.31	**0.01**	0.12	0.08	7.28	0.003*	0.008	0.005	0.48	0.621
5	0.24	0.13	5.39	0.002*	0.16	0.08	7.08	0.004*	0.06	0.03	2.62	0.069	0.005	0.002	0.2	0.886
6	0.22	0.12	5.2	0.002*	0.25	0.14	12.24	0.001*	0.09	0.05	4.25	**0.017**	0.006	0.003	0.31	0.827
7	0.04	0.03	1.09	0.35	0.11	0.08	5.66	0.003*	0.03	0.02	1.42	0.22	0.007	0.005	0.35	0.856
8	0.06	0.04	1.57	0.15	0.01	0.01	0.5	0.71	0.1	0.07	5.41	0.002*	0.015	0.011	0.82	0.491

P values < 0.05 are bolded. P values below the Bonferroni corrected threshold of 0.00625 are indicated with an asterisk.

D, reach direction. F, F statistic. P, p value. SS, sum of squares.

**Fig 2:**
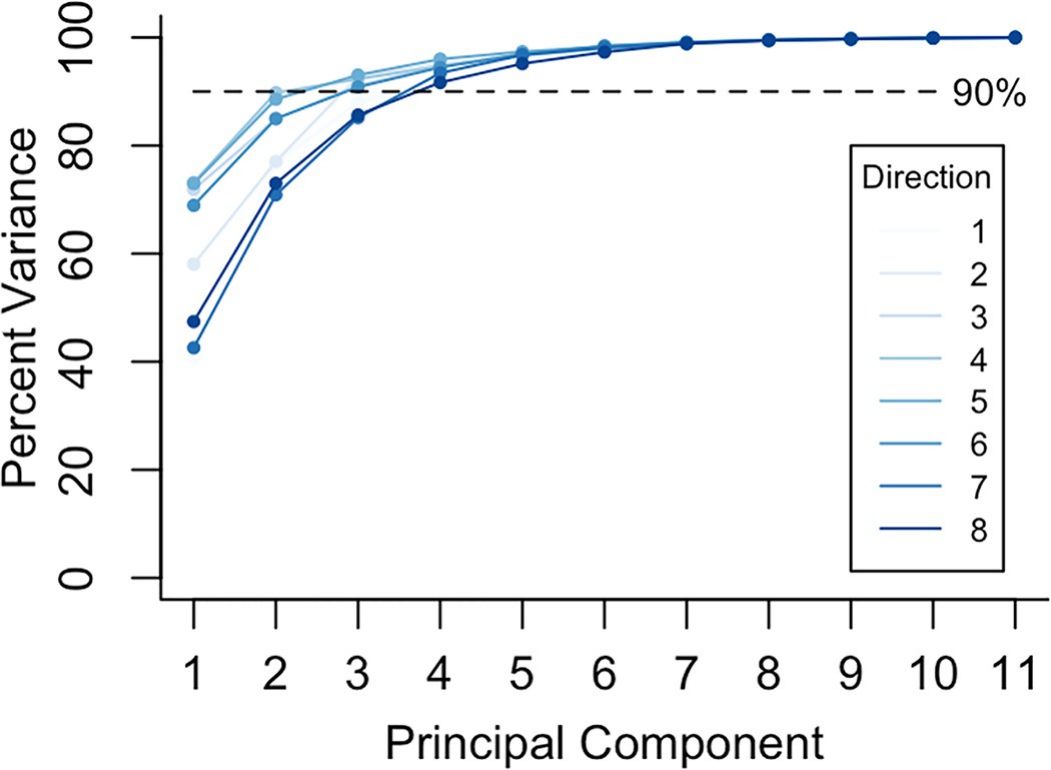
Between Subject Variance Explained by each Principal Component for each Reach Direction. Following principal component analysis, the percentage of overall variance in posture explained by each principal component (i.e. mode of posture variation) was recorded. The first four principal components explained greater than 90% of the variance for each reach direction.

To further investigate these differences in posture following GPA, principal component analyses (PCA) were performed on the posture shape coordinates (i.e. joint centers) in 11-dimensional tangent space, with each principal component (PC) representing a “mode” of posture variation. Four out of the 11 PC vectors accounted for greater than 90% of the overall variance in posture between subjects at the time of maximal reach in each of the eight reach directions (Fig 2). For each subject, posture at the time of maximal reach was represented as a linear combination of the four PCs explaining the highest proportion of variance in each direction. In an analysis of variance, there were significant relationships between subject group and PC loading in reach one (PC2, p = 0.01; PC3, p = 0.048), reach two (PC1, p = 0.0032; PC2, p = 0.0085), reach three (PC1, p = 0.0013; PC2, p = 0.045), reach four (PC1, p = 0.0042; PC2, p = 0.0022), reach five (PC1, p = 0.0093; PC2, p = 0.0021) and reach six (PC1, p = 0.0082; PC2, p = 0.018). There was no association between subject group and PC loading in reach seven (Table 4).

**Table 4:** Comparison of posture variation between groups at the time of maximal reach.

Direction	ANOVA P Values				PC 1: T Test vs Controls		PC 2: T Test vs Controls	
	PC1	PC2	PC3	PC4	OA	Post-op	OA	Post-op
1	0.073	**0.01**	**0.048**	0.82			**0.028**	0.58
2	0.0032*	0.0085*	0.14	0.25	< 0.001*	0.94	**0.011**	0.17
3	0.0013*	**0.045**	0.96	0.13	< 0.001*	0.45	0.0029*	0.54
4	0.0042*	0.0022*	0.33	0.31	0.00023*	0.76	< 0.001*	0.74
5	0.0093*	0.0021*	0.14	0.061	0.0011*	0.93	< 0.001*	0.1
6	0.0082*	**0.018**	0.87	0.93	0.0011*	0.2	0.0019*	0.53
7	0.37	0.39	0.62	0.25				
8	0.097	0.9	0.14	0.79				

P values < 0.05 are bolded. P values below the Bonferroni corrected threshold of 0.00625 are indicated with an asterisk. ANOVA, analysis of variance. PC, principal component.

**Fig 3:**
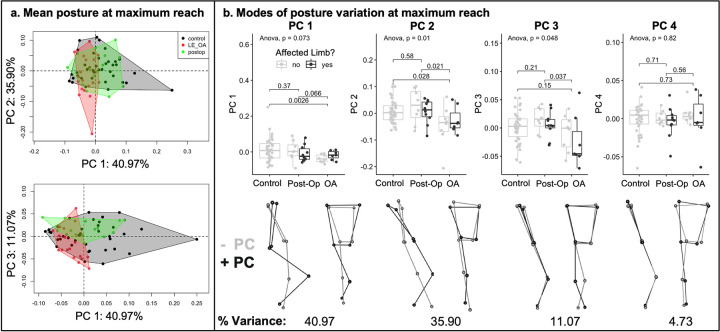
Principal Component Analysis of Postures at Maximal Reach. Principal component analysis was performed on maximum reach posture in each of the eight reach directions. Data are presented here for the anterior reach direction (direction one). a. The posture of each subject at maximum reach is plotted in principal component space along PC1 and PC2 (top) as well as PC1 and PC3 (bottom). Each point on the graph represents a posture. Black circles represent healthy controls. Green circles represent asymptomatic postoperative patients. Red circles represent symptomatic osteoarthritis patients. b. Histograms depict raw principal component values for each subject grouped according to cohort. Error bars represent standard error. The first four modes of posture variation at the time of maximum reach are displayed to visualize the results of the principal component analysis. Dark grey skeletons represent maximum values for that particular mode of variance and light grey skeletons represent the minimum values.

Direct comparisons of the modes of posture variation of OA patients and asymptomatic postoperative patients against the control group were performed using t tests when the ANOVA result was significant. PC loading in the OA cohort was significantly different than the control group in six of the eight reach directions (Table 4). For example, in reach one, patients with OA had significantly lower contributions from PC 2 (35.90% of overall variance) and PC 3 (11.07% of overall variance) than controls (0.028, and 0.037 respectively). The higher contributions from PC 2 among the control group represented greater knee flexion with increased spine extension (Fig 3). Lower values of PC 3 observed in the OA group were associated with increased knee valgus. In contrast to the OA cohort, no differences were observed between asymptomatic postoperative patients and controls in any reach direction for the two PC’s explaining the highest proportion of posture variance (Table 4).

### Distinguishing subject groups based on time-series postural motion patterns

**Fig 4:**
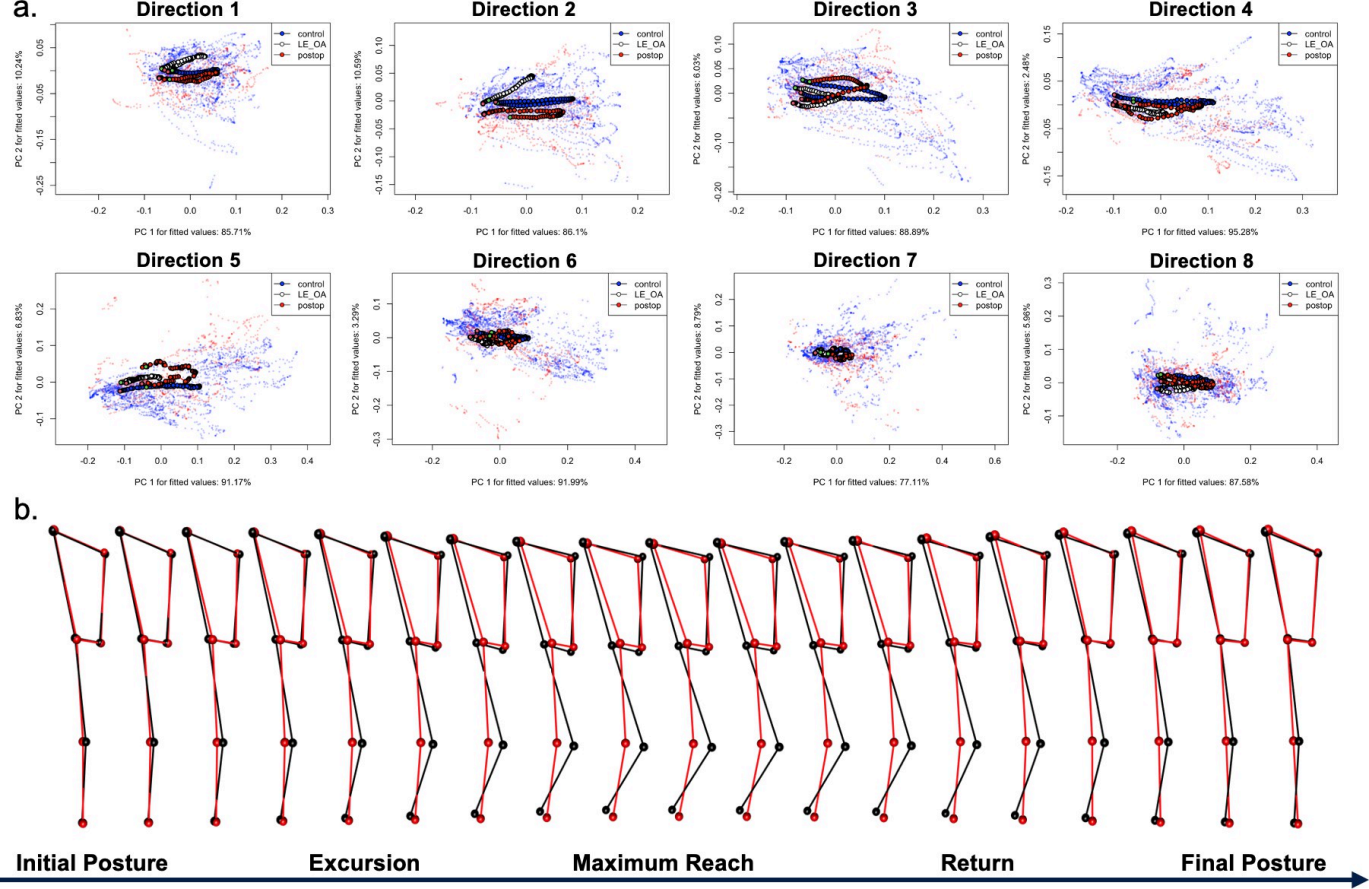
Reach Trajectories by Disease State. a. Reach trajectories are displayed in principal component space for each of the eight reach directions. Each point on the graph represents an entire posture. Each line represents a sequence of postures (i.e. trajectory). Trajectory data for each time point for each subject is plotted in light blue for controls, white for lower extremity osteoarthritis, and red for asymptomatic postoperative patients. The mean trajectory for each group is plotted in dark blue, white, and red for controls, lower extremity osteoarthritis, and for asymptomatic postoperative patients respectively. b. Every third posture along the mean trajectory for the healthy controls (black) and symptomatic osteoarthritis (red) cohorts are plotted in three-dimensional space along the time axis for the second reach direction.

In order to investigate the relationship between time, posture, and disease state, motion during each reach was first represented as ordered sequences of postures through shape space. Mean trajectories for each group, as well as individual subject trajectories, were projected onto the first two PC’s explaining the highest proportion of variance and are displayed in Fig 4. Path distance (total amount of posture change), path shape (how posture changed), and path orientation (the angle between first PC’s of posture trajectory) were compared between disease states for each reach direction using Mantel tests due to their high dimensionality. Path distance was significantly shorter in the OA group than the control group in reach two (0.18 vs 0.29, p = 0.006), reach three (0.20 vs 0.36, p = 0.004), reach four (0.22 vs 0.41, p = 0.009), and reach five (0.24 vs 0.40, p = 0.037). There were no significant differences in path length between the asymptomatic postoperative patients and the control group.

Compared to control subjects, path shape as measured by the Procrustes distance in shape space was significantly different in the OA patients in reach three (Procrustes distance = 0.45, z = 2.16, p = 0.003) and reach eight (Procrustes distance = 0.68, z = 1.75, p = 0.040). The posture trajectories of asymptomatic postoperative patients were also different than controls in reach two (Procrustes distance = 0.46, z = 2.04, p = 0.015), reach three (Procrustes distance = 0.31, z = 1.84, p = 0.027), and reach five (Procrustes distance = 0.59, z = 1.92, p = 0.023). There were no differences in other reach directions between groups.

Path orientation was significantly different in the OA cohort compared to the controls in reach one (angle = 19.4 deg, z = 1.73, p = 0.036), reach two (angle = 40.4 deg, z = 4.24, p = 0.001), reach three (angle = 27.0 deg, z = 4.16, p = 0.001), and reach four (angle = 18.16 deg, z = 2.44, p = 0.007). Path orientation differed between asymptomatic postoperative patients and controls only in direction three (angle = 16.60, z = 2.65, p = 0.004). There were no significant differences in other reach directions between groups.

### Distinguishing subject groups based on kinematic deviation index and its relationship with patient-reported health status

**Fig 5:**
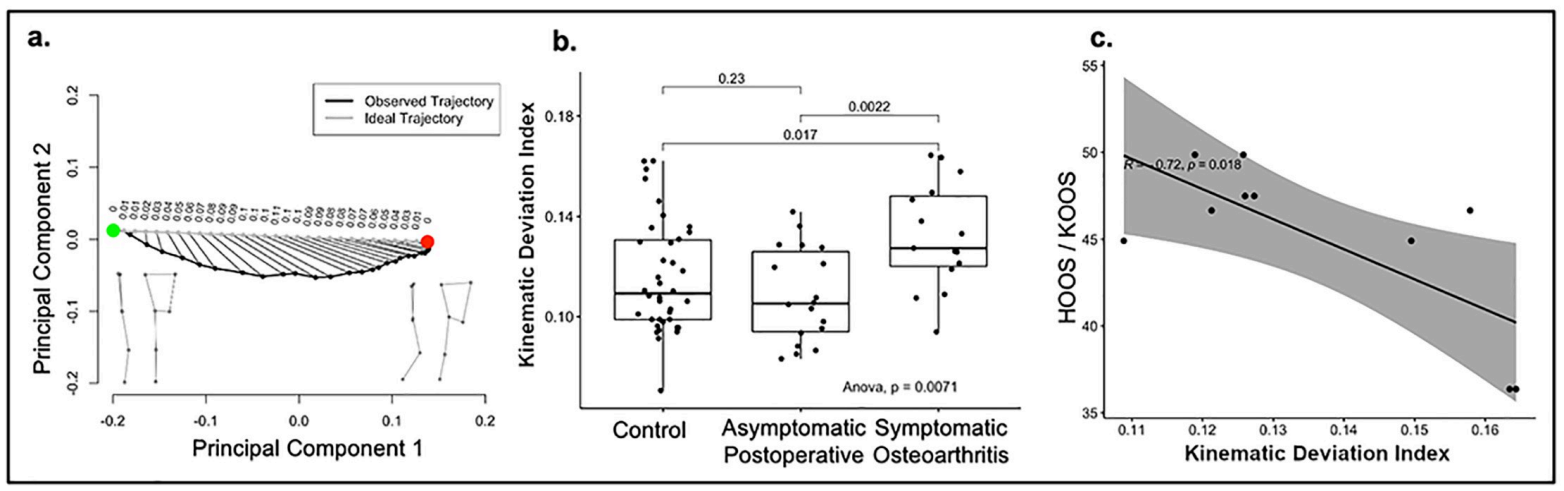
Computation of Kinematic Deviation Index and Correlation with Patient Reported Health Measures. a. Observed versus ideal trajectories for a representative single subject during a single reach. The black line represents the observed trajectory plotted in principal component space. The grey line represents the theoretical “ideal trajectory” (i.e. straight line through tangent space). The green point represents the initial posture and the red point represents the posture at maximum reach. The actual subject postures are reconstituted in three dimensions for the initial and maximal reach postures. b. Kinematic deviation index plotted by disease state. Histograms depict raw values for each subject grouped according to cohort. Error bars represent standard error. C. Correlation of kinematic deviation index with the patient reported health measures, hip disability and osteoarthritis outcome score (HOOS) and knee injury and osteoarthritis outcome score (KOOS).

Kinematic deviation index (KDI) was developed as a method to quantify dynamic postural control during the assessment. For each subject, KDI was calculated by comparing the subject’s observed posture trajectory in 11-dimensional tangent space to a subject-specific theoretical trajectory with the least overall joint motion (Fig 5). One KDI score is reported for each patient, which represents KDI averaged over all eight reach distances. In an analysis of variance, there was a significant association between groups and KDI (F = 6.56, df = 68, ANOVA p = 0.0071). In direct comparisons using t tests, patients with OA (mean = 1.69, SD = 0.49) had significantly greater KDI than both heathy controls (mean = 1.23, SD = 0.40) (t = - 3.19, df = 21.52, p = 0.0043) and asymptomatic postoperative (mean = 1.27, SD = 0.41) (t = -2.60, df = 27.4, p = 0.015) patients.

Within the OA cohort, a significant correlation was observed between KDI and patient-reported Hip Disability and Osteoarthritis Outcome Score and Knee injury and Osteoarthritis Outcome Score scores (R = - 0.72, p = 0.018).

## Discussion

The results of this analysis demonstrate enhanced clinical utility of time series motion capture data compared to conventional functional tests in the case of the SEBT. First, the accuracy of MMC for recording conventional SEBT reach distances in a clinic setting was validated against manual measurements performed in the standard fashion. Then in a cohort of healthy controls, symptomatic OA patients, and asymptomatic postoperative patients, it was shown that the conventional SEBT reach distances poorly distinguished between groups. However, both time series and static three-dimensional shape models of joint position of the stance leg and trunk did reliably distinguish between groups in most reach directions. Finally, the KDI was proposed to summarize subject performance, which distinguished between disease states and correlated with patient reported outcomes in the cohort of OA patients.

Human motion is the product of complex coordination between the central and peripheral nervous systems and the musculoskeletal system. Because it is currently not possible to observe these interactions directly, existing functional tests rely on proxy endpoint data such as reach distances to assess these underlying systems. In the case of the SEBT, conventional reach distances are a proxy for dynamic postural control. Neuromuscular and musculoskeletal pathologies, as well as subject characteristics impact the body’s ability to maintain balance under stress and can affect the observed reach distances during SEBT.

While these proxy endpoints allow for more pragmatic implementation of functional tests in clinic, they are prone to error in their oversimplification of the underlying physiology, and as a result, may miss subtle manifestations of disability. The use of three-dimensional motion trajectory data offers more comprehensive and relevant endpoints. In the case of SEBT, reach distances do not account for the potential impact of alternate reach strategies or compensatory motions to maintain balance. For example, while a prior study found no difference in SEBT reach distances between patients with chronic ankle instability and those without, they noted significantly different pelvis and trunk rotation at maximal reach [22]. Similarly, Robinson showed that SEBT reach distances are largely a function of stance leg kinematics, which are not routinely assessed in clinic [23]. Our results further these previous studies and suggest that even in cases where there is no difference in conventional SEBT reach distances between groups, significant differences still persist in three-dimensional posture and time series motion.

Procrustes shape analyses have been used previously with biomechanical data. For example, Wang used a two-dimensional Procrustes shape model to develop a gait recognition algorithm based on the silhouettes of ambulating subjects [24]. While other quantitative methods of distinguishing movement strategies have been employed [25], Adams and Cerney were the first to apply Procrustes shape modeling to three-dimensional joint position motion data, distinguishing squat lifting and stoop lifting in a series of subjects [26].

A key advantage of MMC with shape modeling over conventional functional tests is the ability to assess relationships between disease state, age, sex, and body characteristics and movement strategies. This has previously been documented in the upper extremity, where sex and age influence movement strategies in subjects reaching towards fixed targets [27]. A prior exploratory factor analysis identified leg length and height as predictors of SEBT reach distances, but found no association between reach distances and sex [28]. Our analysis similarly found no association between sex and reach distance. However, there was a significant relationship between sex and posture at maximal reach in five of the eight reach directions. While males and females may achieve similar reach distances, they may employ different reach strategies based on differences in bone shape, muscle strength, and other parameters. With regards to age, prior studies have suggested younger subjects may reach farther than older subjects [29]. Our study extends prior findings to suggest that while age is not only associated with reach direction, it is also associated with posture at maximal reach. This suggests age-related changes in musculoskeletal physiology and motor control may influence participants to alter their reaching strategies.

Prior attempts have been made to create a composite score for the SEBT, which has generally been described as an average of reach distances [8, 30]. Although this score provides a pragmatic method of comparing overall performance between subjects, the reach distance itself is still only a proxy for motor control and does not reflect the results of an underlying biomechanical assessment. The introduction of KDI captures the results of an advanced analysis of three-dimensional motion trajectories in a single numerical score. Subjects performing the SEBT using controlled movements with minimal off target motion travel along a path in shape space more similar to the theoretical ideal motion trajectory. In our analysis, KDI was more discriminative between groups than conventional reach distances, with OA patients demonstrating significantly greater KDI. A recent review found that movement variability during performance of dynamic activities is significantly different in patients with musculoskeletal injury compared to those without, with a trend towards greater movement variability in injured groups [31]. Interestingly, there was no difference in KDI between asymptomatic postoperative patients and healthy controls, suggesting symptom severity may be related to SEBT performance. There was also a correlation between KDI and HOOS and KOOS scores among the OA patients, suggesting that patients who deviate farther from the ideal trajectory during SEBT also subjectively experience worse symptoms.

Current functional tests and movement screens are poor predictors of lower extremity injury risk, and there is a significant need for cohort studies investigating new risk assessment tools [11, 32]. As prior studies have shown SEBT performance to be associated injury risk, future investigation of KDI as a screening tool could be warranted [13]. SEBT has also been validated as a tool to track progress in various lower extremity injury patient populations during therapy [8, 33, 34]. The approach described in this analysis can be practically implemented in most clinics and would provide a quantitative and objective assessment of performance that can be monitored over time. The COVID-19 pandemic has highlighted opportunities for technology to augment existing rehabilitation programs and to offset associated costs, especially in the arthroplasty population [35, 36]. Given the low cost and simple configuration of MMC systems, it is feasible for in home and remote deployment, as has been proposed with other systems [37].

This main strengths of this analysis are a pragmatic application of three-dimensional motion analysis in a clinical setting as well as the introduction of a novel KDI score to capture lower extremity postural control. Our analysis of the posterolateral reach directions where subjects reach behind their stance leg was limited by auto-occlusion of the reaching leg by the stance leg. This is a limitation of the machine learning methods used by Microsoft’s body tracking API and reflect an inherently difficult task with a single camera set up. While the addition of more cameras could alleviate this problem, it would decrease the practicality of using this method in clinic. These posterolateral reach movements rarely occur in most patient populations, including most sports. Finally, given that there may be some redundancy in the eight reach directions, a reduction of the number of reaches per trial is likely justified and could simplify future data collection.

In conclusion, we present a robust and accessible method for capturing three-dimensional motion data of the lower extremity, demonstrate its utility in distinguishing patient populations, and show the relationship between our analysis and standard patient reported health measures. Future analyses should focus on the use of these methods in injury risk assessment and the longitudinal monitoring of patient responses to surgical and nonsurgical interventions.

## Materials and methods

### Study design and population

All study protocols and recruitment were approved by the University of California San Francisco Human Research Protection Program. Patients older than 18 years of age were recruited from routine visits to an ambulatory care center for participation in a clinic-based motion analysis session. Informed consent was obtained from each participant. Patients were excluded from the study if any assistive device was required for ambulation or if study personnel determined they were at high risk for a fall based on clinical judgement. Subjects included in the control cohort reported no history of lower extremity pathology requiring treatment (e.g. surgery, nonoperative management). Patients included in the lower extremity OA cohort were receiving treatment for symptomatic hip or knee arthritis, but had no history of joint arthroplasty. Finally, patients with a history of lower extremity orthopaedic surgery who were asymptomatic at the time of the participation were included in a separate group.

### Experimental configuration and data collection

The standard SEBT grid with markings was applied to the floor of a four-by-four meter clinic space with a plain background. During each trial, patients were instructed to reach maximally in each of the eight directions while remaining stable on the stance leg. At the point of maximal reach, patients were instructed to contact the ground with their reaching toe without transitioning weight. Subjects began with the anterior reach direction (direction one), and then proceeded clockwise for right foot reach trials and counter-clockwise for left foot reach trials. Subjects performed two warm up trials on each leg prior to three recorded trials on each leg, and were given unlimited rest between trials. Subjects were instructed to maintain their hands on their hips to minimize use of the arms for balance. If a patient became unstable during a trial, the recording was stopped and the trial was repeated.

A single noninvasive, markerless three-dimensional depth camera (Microsoft Kinect V2, Microsoft, Redmond, WA) was positioned 250 centimeters anterior to the center of the grid at a height of 75 centimeters. The depth camera recorded the positions of the bilateral shoulders, hips, knees, and ankles at a rate of 30 frames per second. Raw joint position data were filtered with a second order low-pass Butterworth filter with a cutoff frequency of three Hz and an allometrically scaled, patient-specific rigid body model [4]. Reach distances were also manually recorded in centimeters as the distance from the stance leg toe to the reach leg toe. To identify the center of the grid using the depth camera, the translation from the center of the grid to the stance ankle were noted during recording, and corresponding adjustments were made during processing based on the averaged location of the stance ankle for each recording. For subjects with lower extremity OA, HOOS or KOOS was recorded [38, 39].

### Three-dimensional statistical shape modeling procedure

Filtered joint position data for the bilateral shoulders and hips, and stance leg knee and ankle (six total landmarks) for each trial were transformed in a generalized Procrustes analysis (GPA). Since the SEBT is designed to stress the dynamic postural control systems of the stance leg, the reaching leg knee and ankle were not included in this analysis. GPA is the primary method of comparing shape variables from landmark coordinates used in geometric morphometrics [40, 41]. In this technique, the three-dimensional joint position data are scaled, rotated, and translated mathematically to minimize the distance between corresponding landmarks between subjects (Fig 1). Although the dimensionality of the raw subject data is 18 (six landmarks recorded in three dimensions), the aligned Procrustes coordinates following GPA are present in 11-dimensional curved shape space [42]. Seven degrees of freedom are lost during standardization [40]. Data are subsequently projected from curved shape space into Euclidean tangent space for statistical analysis without any additional loss of dimensionality. Data were filtered using MATLAB (*The MathWorks Inc*, *Natick*, *MA*) and GPA was performed in R using the *Geomorph* (Version 4.0) and *RRPP* Packages (Version 0.602).

### Assessing accuracy of conventional SEBT using MMC

All reach distances were normalized to subject leg length, as measured from the anterior superior iliac spine to the medial malleolus, as this has previously been shown to correlate with reach distance [28]. The correlations between manual and depth camera reach distance measurements in each reach direction were assessed using repeated measures correlations (S1 Data, S1 Text). In contrast to the manual measurements which were recorded from the origin to the reach toe, depth camera measurements were recorded from the origin to the reach ankle, due to the higher fidelity of the ankle joint position data compared to the foot marker. The expected offset was confirmed using Bland-Altman plots.

### Distinguishing subject groups using conventional SEBT

Depth camera reach distances were compared between groups using repeated-measures, mixed methods linear regression models. Repeated measures within subjects (e.g. multiple trials of the same reach direction) were modeled as random intercepts and age, sex, and affected limb status were modeled as fixed effects. P values were estimated using the Satterthwaite’s approximation, as this has been shown to produce acceptable type I error. Analysis of reach directions was performed using R (R Foundation, Vienna, Austria).

### Distinguishing subject groups using three-dimensional posture at maximal reach

The time of maximal reach was selected for analysis since it corresponds to the conventional SEBT output, reach distance. The first trial for each stance leg per subject was selected for analysis to minimize the potential effect of fatigue on posture. The three-dimensional posture of each subject at maximal reach (i.e., matrix of coordinates of the bilateral shoulders, hips, and stance leg knee and ankle) was recorded after alignment in a generalized Procrustes analysis. To assess the relationship between posture at maximal reach and disease state, Procrustes linear models were generated for each direction of the SEBT controlling for effects of age, sex, and primarily affected leg. Procrustes linear models are fit to the superimposed postures using maximum likelihood estimation on the sum-of-squared Procrustes distances through a residual randomization permutation procedure [43, 44].

To further investigate between group differences in posture at the time of maximal reach, principal component analysis (PCA) was performed on Procrustes shape coordinates in the tangent plane. Since the Euclidean tangent space is of 11 dimensions, there are 11 principal components for each posture. For each reach direction, the percent of total variance in posture explained by each PC was recorded. Individual subject data were plotted in PC space along the first and second, and first and third principal components containing the highest percentage of variation. Overall group differences in loading for the first four PC’s were compared using an ANOVA. The OA and post-operative group loadings were compared directly to controls using T tests if the ANOVA p value was significant. To facilitate interpretation of the PCA results, the minimum and maximum loadings along each of the first four principal components were reconstituted from principal component space to three-dimensional posture space (Fig 3).

### Distinguishing subject groups based on time-series postural motion patterns

SEBT trials were represented as ordered sequences of postures in shape space over time (Fig 4). Since posture shapes were standardized for size, translation, and rotation in GPA, trajectories represent change in posture during each SEBT reach. As the SEBT was self-paced, motions were defined temporally as the 30 frames prior to and 30 frames following the time of maximal reach in each direction, resulting in 60 frames per trajectory. Three trajectory characteristics were compared between groups: path distance (the extent to which posture changed over each trial), shape (how posture changed during each trial), and orientation (the angle between first principal components of trajectories for each trial). Due to the high dimensionality of the trajectory data (60 observations of six tracked joints in three dimensions), the distance, shape, and orientation of trajectories were compared using Mantel tests [45].

### Distinguishing subject groups based on Kinematic Deviation Index and its relationship with patient-reported health status

KDI was developed to quantify postural control during the entire SEBT assessment. Mathematically, KDI represents the amount to which a posture trajectory deviates from a theoretical ideal trajectory during a movement. In tangent space, the shortest trajectory from one posture (e.g. rest) to another posture (e.g. maximal reach) is a straight line. Although this path may not represent the path of minimum physiologic energy expenditure, it does represent the theoretical path with the minimum necessary amount of posture change. During motion, deviation from the ideal trajectory occurs when multiple types of posture change occur or when the rate of posture change is variable [46]. To calculate KDI, posture trajectories from resting posture to the point of maximal reach for each reach direction for each subject were identified and transformed using GPA to standardize for shape, translation, and rotation. For each trial, an ideal trajectory was defined as the straight line connecting the rest posture to the point of maximal reach in tangent space. The distances between corresponding time points on the ideal and observed trajectories were calculated for each frame. KDI for each reach was defined as the sum of the squares of these distances normalized by trajectory length (e.g. the total amount of postural change) so as to not penalize subjects undergoing more posture change. Finally, the mean KDI over each of the eight reach directions was reported as the overall KDI for the entire trial.

Regarding interpretation, subjects with higher KDI scores deviated more from the theoretical trajectory (e.g. exhibited multiple types of shape change or temporal variability) and therefore exhibited less postural control. Overall KDI was compared between groups using ANOVA. To assess the relationship between KDI and HOOS and KOOS, Pearson’s correlation coefficients were employed.

## Supporting information

S1 Data Reach Distance Data for Each Subject. For each participant, S1 Data contains the manually measured and motion analysis derived reach distances, as well as laterality. This data can be used with S1 Text to compare reach distances between groups.
(XLSX)

S1 Text Code for Processing Reach Distances. S1 Text contains the code (in the R language) used to compare reach distances between groups. For each participant, S1 Data contains the manually measured and motion analysis derived reach distances, as well as laterality.
(DOCX)
