# Supplementary material for: Point-of-care motion capture and biomechanical assessment improve clinical utility of dynamic balance testing for lower extremity osteoarthritis
Source: PLOS Digit Health. 2022 Jul 7;1(7):e0000068. doi: 10.1371/journal.pdig.0000068 (PMC9931224; doi:10.1371/journal.pdig.0000068)
Supplement: S1 Text — contains the code (in the R language) used to compare reach distances between groups. For each participant, S1 Data contains the manually measured and motion analysis derived reach distances, as well as laterality. (DOCX) [file pdig.0000068.s002.docx]

library(rmcorr)

library(readxl)

library(blandr)

library(ggplot2)

library(lme4)

library(lmerTest)

df <- read.csv('rd_df.csv')

x<-rmcorr(sub_lat, k1, m1, df)

print(paste0(round(x$r, digits = 2), ', 95% CI ', round(x$CI[1], digits = 2), '-', round(x$CI[2], digits = 2)))

x<-rmcorr(sub_lat, k2, m2, df)

print(paste0(round(x$r, digits = 2), ', 95% CI ', round(x$CI[1], digits = 2), '-', round(x$CI[2], digits = 2)))

x<-rmcorr(sub_lat, k3, m3, df)

print(paste0(round(x$r, digits = 2), ', 95% CI ', round(x$CI[1], digits = 2), '-', round(x$CI[2], digits = 2)))

x<-rmcorr(sub_lat, k4, m4, df)

print(paste0(round(x$r, digits = 2), ', 95% CI ', round(x$CI[1], digits = 2), '-', round(x$CI[2], digits = 2)))

x<-rmcorr(sub_lat, k5, m5, df)

print(paste0(round(x$r, digits = 2), ', 95% CI ', round(x$CI[1], digits = 2), '-', round(x$CI[2], digits = 2)))

x<-rmcorr(sub_lat, k6, m6, df)

print(paste0(round(x$r, digits = 2), ', 95% CI ', round(x$CI[1], digits = 2), '-', round(x$CI[2], digits = 2)))

x<-rmcorr(sub_lat, k7, m7, df)

print(paste0(round(x$r, digits = 2), ', 95% CI ', round(x$CI[1], digits = 2), '-', round(x$CI[2], digits = 2)))

x<-rmcorr(sub_lat, k8, m8, df)

print(paste0(round(x$r, digits = 2), ', 95% CI ', round(x$CI[1], digits = 2), '-', round(x$CI[2], digits = 2)))

rd_mixed = lmer(k5 ~ group + (1 | sub_lat), data = df)

summary(rd_mixed)

df_pre <- read_xlsx('rd_df2.xlsx')

df_data <- read_xlsx('final_population_list.xlsx')

df_pre$group1 <- ''

df_pre$group2 <- ''

df_pre$group3 <- ''

df_pre$sex <- ''

df_pre$age <- ''

df_pre$affected <- ''

for (i in 1:length(df_pre$participants_by_trial)){

df_pre$sex[i] <- df_data$Sex[which(df_pre$participants_by_trial[i] == df_data$Subject & df_pre$lat_t[i] == df_data$Laterality)]

df_pre$age[i] <- df_data$Age[which(df_pre$participants_by_trial[i] == df_data$Subject & df_pre$lat_t[i] == df_data$Laterality)]

df_pre$affected[i] <- df_data$isAffected[which(df_pre$participants_by_trial[i] == df_data$Subject & df_pre$lat_t[i] == df_data$Laterality)]

df_pre$group1[i] <- df_data$Groups1[which(df_pre$participants_by_trial[i] == df_data$Subject & df_pre$lat_t[i] == df_data$Laterality)]

df_pre$group2[i] <- df_data$Groups2[which(df_pre$participants_by_trial[i] == df_data$Subject & df_pre$lat_t[i] == df_data$Laterality)]

df_pre$group3[i] <- df_data$Groups3[which(df_pre$participants_by_trial[i] == df_data$Subject & df_pre$lat_t[i] == df_data$Laterality)]

}

df_post <- df_pre

write.csv(df_post, "df_post.csv")

rd_mixed = lmer(as.numeric(k7) ~ group3 + as.numeric(age) + sex + affected + (1 | sub_lat), data = subset(df_post, df_post$group3 != "exclude"))

summary(rd_mixed)

confint(rd_mixed)
